# Identification of Glucose-Regulated Protein 78 (GRP78) as a Receptor in BHK-21 Cells for Duck Tembusu Virus Infection

**DOI:** 10.3389/fmicb.2018.00694

**Published:** 2018-04-09

**Authors:** Dongmin Zhao, Qingtao Liu, Kaikai Han, Huili Wang, Jing Yang, Keran Bi, Yuzhuo Liu, Na Liu, Yujie Tian, Yin Li

**Affiliations:** Institute of Veterinary Medicine, Jiangsu Academy of Agricultural Sciences, Key Laboratory of Veterinary Biological Engineering and Technology, Ministry of Agriculture, Nanjing, China

**Keywords:** tembusu virus, BHK-21 cells, glucose-regulated protein 78, virus entry, receptor

## Abstract

Since 2010, outbreak and spread of tembusu virus (TMUV) caused huge losses to the breeding industry of waterfowl in several provinces of China. In this study, we identify the glucose-regulated protein 78 (GRP78) as a receptor in BHK-21 cells for duck TMUV infection. Using cell membrane from BHK-21 cells, a TMUV-binding protein of approximately 70 kDa was observed by viral overlay protein binding assay (VOPBA). LC-MS/MS analysis and co-immunoprecipitation identified GRP78 as a protein interacting with TMUV. Antibody against GRP78 inhibited the binding of TMUV to the cell surface of BHK-21 cells. Indirect immunofluorescence studies showed the colocalization of GRP78 with TMUV in virus-infected BHK-21 cells. We found that GRP78 over-expression increased TMUV infection, whereas GRP78 knockdown by using a specific small interfering RNA inhibited TMUV infection in BHK-21 cells. Taken together, our results indicate that GRP78 is a novel host factor involved in TMUV entry.

## Introduction

Tembusu virus (TMUV) caused severe disease in ducks and spread rapidly in many duck-producing provinces of China since 2010. The morbidity reaches 100% and the mortality ranges from 5 to 30%. It is reported that domestic chicken is sensitive to this newly emerging disease and exhibits similar clinical signs (Liu et al., 2012; Tang et al., 2012; Chen et al., 2014). Recent studies indicate that TMUV can be isolated from other birds like geese, pigeons as well as house sparrows (Liu et al., 2012; Tang et al., 2013; Dai et al., 2015). TMUV has become one of the widespread infectious diseases of ducks, which lead to serious economic loss in duck industry of China (Wang et al., 2011).

Tembusu virus, family Flaviviridae, genus *Flavivirus*, is made up of three structural proteins (envelope [E], capsid [C], and membrane [M]) and seven non-structural proteins (NS1, NS2a, NS2b, NS3, NS4a, NS4b, and NS5). Among these viral proteins, E protein is the main structural protein, which has three separate domains and form head-to-tail homodimers on the surface of the virion (Oliphant et al., 2006). The E protein of flavivirus mainly functions as receptor binding protein, which is the primary determinant of virulence, cell tropism, and host range. Antigenic analysis shows that E protein contains many epitopes which elicit neutralizing antibodies during the immune response (Roehrig, 2003; Chu et al., 2005). Besides, E protein mediates fusion between virus and host membrane in the acidic condition of the late endosomes (Heinz and Allison, 2003; Chu and Ng, 2004a).

As enveloped virus, the first step in the infection of flavivirus involves the binding between E protein and cellular receptor of host cell. Flavivirus can recognize uniquitous cell surface molecules or utilize multiple receptors for cell entry (Rodenhuis-Zybert et al., 2010). Subsequently, flaviviruses enter cells through clathrin-mediated endocytosis. After clathrin-mediated entry, virion is delivered to endosomes. The acidic condition of endosome triggers E protein mediated membrane fusion between viral and endosomal membrane. Finally, fusion pore is formed and nucleocapsid is released into the cytosol (Smit et al., 2011).

In recent years, several binding factors have been identified, suggesting that flavivirus may use multiple receptors for cell entry, which is strain-specific and/or cell type-dependent (Medigeshi et al., 2008). C-type lectin expressed by monocyte-derived dendritic cells, named DC-SIGN or CD209, mediates dengue virus (DENV) infection. DC-SIGN is considered to be one of the most important receptors for DENV (Navarro-Sanchez et al., 2003; Tassaneetrithep et al., 2003; Lozach et al., 2005; Alen et al., 2009). Mannose receptor (MR, CD206) in macrophages binds to all four serotypes of DV and specifically to the E protein (Miller et al., 2007). Heparan sulfate expressed by almost all cell types, is a non-specific receptor molecule responsible for DENV attachment in several cell lines (Chen et al., 1997; Dalrymple and Mackow, 2011). Cellular α_v_β_3_ integrin, laminin-binding protein and C-type lectin were reported to function as West Nile virus (WNV) receptors (Chu and Ng, 2004b; Bogachek et al., 2010; Cheng et al., 2010). Heat shock protein 90 (HSP90), HSP70, vimentin, laminin receptor, CD4, α_v_β_3_ integrin and DC-SIGN played a role in Japanese encephalitis virus (JEV) entry (Chu and Ng, 2004b; Ren et al., 2007; Das et al., 2009; Thongtan et al., 2012; Nain et al., 2016). Recent studies identified HSPA9 as a TMUV binding protein in DF-1 cells (Liu et al., 2017). However, it is still unclear about TMUV-binding molecules in BHK-21 cells.

In the present study, glucose-regulated protein 78 (GRP78) was identified as a cellular molecules that involves in TMUV infection in BHK-21 cells using viral overlay protein binding assay (VOPBA) technique and mass spectroscopy. Our results demonstrated that GRP78 played important role in TMUV entry into the cell.

## Materials and Methods

### Cells, Viruses, and Antibodies

BHK-21 cells were maintained in RPMI 1640 medium supplemented with 10% inactivated fetal calf serum (FCS) plus 100 μg/ml of penicillin/streptomycin at 37°C in 5% CO_2_ (Zhao et al., 2015b). TMUV JS804 was isolated from an affected goose with neurological clinical signs, propagated in BHK-21 cells and used in this study (Huang et al., 2013).

Monoclonal antibody against JS804 E protein was produced by this laboratory (Niu et al., 2013). Alkaline phosphatase-conjugated goat anti-mouse IgG and DAPI were purchased from the Beyotime Institute of Biotechnology. GRP78 N-terminal (ab32618, rabbit), GRP78 C-terminal (ab21685, rabbit), goat anti-rabbit IgG (Alexa Fluor 488, ab150077), goat anti-mouse (Alexa Fluor 594, ab150080), and anti-GAPDH antibody (ab8245) were purchased from Abcam. Anti-Na^+^/K^+^ ATPase antibody was purchased from Sigma. HRP-conjugated secondary antibodies were obtained from Invitrogen.

### Viral Overlay Protein Binding Assay

The membrane proteins from BHK-21 cells were isolated using Mem-PER Plus Kit (Thermo) in accordance with the manufacturer’s protocol. VOPBA was performed as essentially described in Thepparit and Smith (2004). Briefly, membrane proteins were subjected to electrophoresis through 12% SDS-PAGE and transferred to PVDF membranes. The membrane containing transferred proteins was blocked with 5% BSA in PBST at 37°C for 2 h. For viral overlay, the membranes were incubated with 10^5^ TCID_50_ of TMUV in 5% BSA in PBST overnight at 4°C and washed three times with PBST buffer. Subsequently, the membranes were incubated with monoclonal antibody against TMUV at 37°C for 1 h followed by incubated with a alkaline phosphatase-conjugated goat anti-mouse IgG. Finally, the signal was developed using Alkaline Phosphatase Assay Kit (Beyotime) in accordance with the manufacturer’s protocol.

### Mass Spectroscopy

To identify viral binding band, the position equivalent to the major virus binding band was excised from duplicated gel and sent for commercial mass spectrometry (LC-MS/MS) analysis. Mass spectrometry was undertaken commercially by Shanghai Luming Biotechnology Company.

### Co-immunoprecipitation Assay

Co-immunoprecipitation assay was carried out as previously described (Lin et al., 2007). Briefly, the membrane proteins from BHK-21 cells were incubated with TMUV on a rocker at 4°C overnight followed by incubation with monoclonal antibody against TMUV for 5 h. Subsequently, protein A/G agarose beads were added to the mixture and incubated for 3 h. The beads were washed by PBS for five times and boiled in 4× SDS-PAGE loading buffer for 5 min. Samples were then analyzed by SDS-PAGE and western blot with anti-GRP78 antibody (ab21685) and HRP-conjugated goat anti-rabbit IgG.

### Inhibition of TMUV Infection by GRP78 Antibody

BHK-21 cells were incubated with 100 μg/ml of anti-GRP78 antibody (ab21685 or ab32618) or rabbit IgG (as negative control) at 4°C for 1 h. Unbound antibodies were removed by washing the cells twice with chilled PBS. The cells were then infected with 200 TCID_50_ TMUV for 30 min at 4°C followed by 1 h at 37°C. Cells were then washed twice with PBS and 1640 containing 1% FCS was added. After 24 h, viral RNA was extracted by QIAamp Viral RNA Mini Kit (QIAGEN) and TMUV RNA levels were determined by qRT-PCR. The primers used for qRT-PCR were described previously (Zhao et al., 2015b): EF primer (forward, 5′-GTGAGATCTTACTGCTATGAG-3′) and the ER primer (reverse, 5′-ACTTGGCACATGTCTGTATGC-3′). qRT-PCR data were analyzed using the comparative C_T_ method (ΔΔC_T_) as described in Zhao et al. (2015a). GAPDH was chosen as a reference gene for internal control. The negative control was used as a reference for each comparison. Differences between the ΔC_T_ of each sample and reference sample [ΔΔC_T_ = (CT_target_–CT_internal control_)sample-(CT_target_–CT_internal control_)negative control] were calculated. Three independent experiments were carried out.

### Western Blot for Surface Expression of GRP78

The membrane proteins and cytosolic proteins from BHK-21 cells were isolated using Mem-PER Plus Kit (Thermo) as described above. Membrane proteins and cytosolic proteins were subjected to electrophoresis through 12% SDS-PAGE and transferred to PVDF membranes. The protein was probed by anti-GRP78 antibody (ab21685).

### Indirect Immunofluorescent Assay

BHK-21 cells grown in 48-well plate were infected with 200 TCID_50_ TMUV. At 24 h post-infection, the cells were fixed with 4% paraformaldehyde and blocked with 5% BSA in PBS. The cells were then incubated with a rabbit anti-GRP78 antibody (ab21685) and monoclonal antibody against TMUV as the primary antibodies followed by an Alexa-488-anti-rabbit and Alexa-594-anti-mouse as the secondary antibodies. Nuclei of the cells were stained with DAPI. The cells were then examined by fluorescence microscope.

### RNA Interference and TMUV Infection

GRP78 shRNA were designed and synthesized by Shanghai Asia-Vector Biotechnology Company with siRNA target site of 5′-GGATTGAGATTGAGTCCTTCT-3′. BHK-21 cells in 6-well plate were, respectively, transfected with GRP78 shRNA and empty pLL3.7-GFP vector by using Lipofectamine-2000 (Invitrogen) according to the manufacturer’s instructions. At 48 h post-transfection, GRP78 mRNA levels were checked by qRT-PCR and cell surface protein was biotinylated and purified. The knock-down of surface-expressed GRP78 was determined by western blotting with GRP78 antibody (ab21685). To determine the TMUV entry in shRNA-transfected cells at 48 h post-transfection, cells were infected with 200 TCID_50_ TMUV at 4°C for 1 h. The cells were washed once with chilled PBS and 1640 containing 10% FCS was added followed by incubated at 37°C for 2 h. Cells were then washed once with chilled PBS and collected. Viral RNA was extracted and determined by qRT-PCR as described above.

### Over-Expression of GRP78 and TMUV Infection

Total RNA was extracted from the BHK-21 cells using TRIzol (Invitrogen) according to the manufacturer’s instructions. cDNA was synthesized by PrimeScript^TM^ 1st Strand cDNA Synthesis Kit (TAKARA). Gene fragment of GRP78 was amplified by PCR using following primers. Forward primer, 5′-GGATCCATG*GACTACAAAGACGACGACGACAAA*AAGTTCACTGTGGT-3′ and reverse primer, 5′-GCGGCCGCCCTA*TTTGTCGTCGTCGTCTTTGTAGTC*AACTCATCTTTTTCTG-3′. The BamHI and *Not* I restriction sites are underlined. The sequence of FLAG tag (DYKDDDDK) is italicized. The PCR product of 2014 bp was digested with the restriction enzymes BamHI and *Not* I and ligated into pcDNA3.1 vector digested with the same restriction enzymes. The resulting construct was designated as GRP78-pcDNA and confirmed by DNA sequencing. BHK-21 cells in 6-well plate were, respectively, transfected with GRP78-pcDNA and empty pcDNA3.1 vector by using Lipofectamine-2000 (Invitrogen) according to the manufacturer’s instructions. At 48 h post-transfection, GRP78 mRNA levels were checked by qRT-PCR and cell surface protein was biotinylated and purified. The over-expression of surface-expressed GRP78 was determined by western blotting with GRP78 antibody (ab21685). To determine the TMUV entry in GRP78-pcDNA-transfected cells at 48 h post-transfection, cells were infected with 200 TCID_50_ TMUV at 4°C for 1 h. Cells were washed once with chilled PBS and 1640 containing 10% FCS was added followed by incubated at 37°C for 2 h. Cells were then washed once with chilled PBS and collected. Viral RNA was extracted and determined by qRT-PCR as described above.

### Cell Surface Protein Biotinylation and Purification

Cell surface protein was biotinylated and purified as described previously (Tsai et al., 2015). The cells were collected and washed three times with chilled PBS to remove contaminating proteins. 0.5 mg/ml EZ-link Sulfo-NHS-SS-Biotin (Thermo) in PBS was added, and cells were gently shaked at 4°C for 30 min. Then biotin solution was removed and Tris-Cl, pH7.5, was added to stop the biotinylation reaction. The cells were rinsed with chilled PBS three times and subject to RIPA lysis (Thermo). To purify surface protein, the lysates were mixed with NeutrAvidin-agarose beads (Thermo) at 4°C overnight. The beads were washed by PBS for five times and boiled in 4× SDS-PAGE loading buffer for 5 min. Samples were then analyzed by SDS-PAGE and western blot.

## Results

### Identification of GRP78 as TMUV-Binding Membrane Protein

**FIGURE 1:**
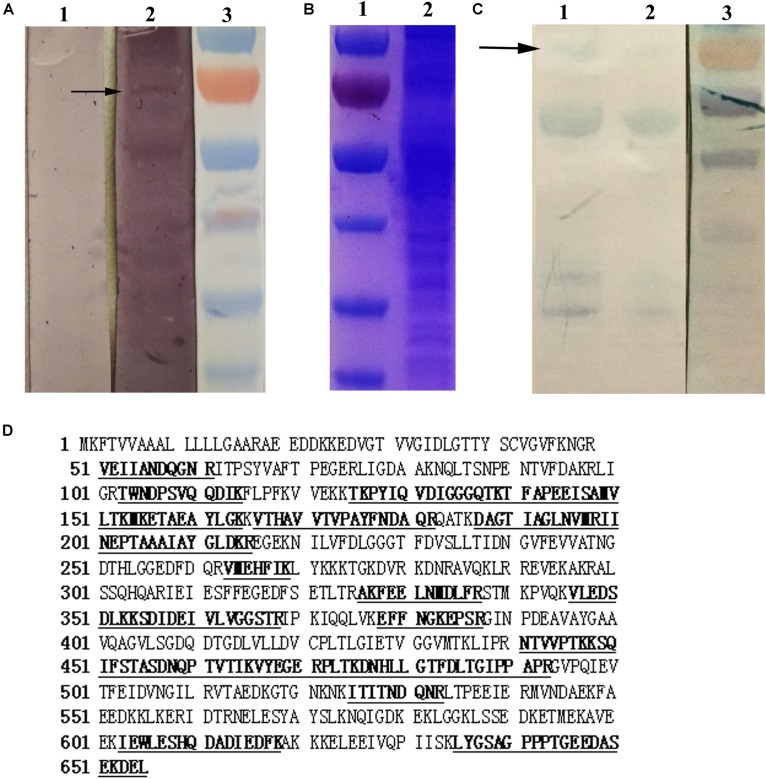
Identification of GRP78 as TMUV-binding membrane protein. **(A)** Detection of protein involved in TMUV binding in BHK-21 cell membrane by VOPBA. The PVDF membrane containing BHK-21 cell membrane proteins were incubated without (Lane 1) or with 10^5^ TCID_50_ of TMUV (Lane 2). Virus binding bands were detected by monoclonal antibody against TMUV. The approximate 70 kDa band was observed in Lane 2 (black arrow). Lane 3, molecular weight marker. **(B)** Coomassie staining of the membrane protein extracted from BHK-21 cells. Lane 1, molecular weight marker; Lane 2, membrane protein extracted from BHK-21 cells. **(C)** Co-immunoprecipitation assay of TMUV binding membrane protein. The membrane protein extracted from BHK-21 cells immunoprecipitated with (Lane 1) or without (Lane 2) TMUV. The immunoprecipitated complexes were analyzed by SDS-PAGE and transferred to PVDF membrane. The membrane was then incubated with anti-GRP78 antibody. The approximate 70 kDa band was observed in Lane 1 (black arrow). Lane 3, molecular weight marker. **(D)** Identification of TMUV binding protein by mass spectrometry. The peptide sequences of GRP78 identified by mass spectrometry were underlined.

Viral overlay protein binding assay was used to preliminarily identify the molecules in BHK-21 cells involved in TMUV binding. A distinct virus binding band of approximately 70 kDa was observed. In absence of TMUV, the monoclonal antibody against TMUV was unable to detect specific binding band (**Figure 1A**). To identify the 70 kDa protein, the protein band equivalent to the major virus binding band was obtained from the gel and sent for commercial mass spectrometry (LC-MS/MS) (**Figure 1B**). The Mascot algorithm was used for databases searches and nine of the most abundant proteins were identified in 70 kDa band (**Table 1**). Of the nine proteins, eight proteins were involved in cell metabolism and cytoskeleton. Since glucose-regulated protein 78 (GRP78) has been identified as receptor of Japanese encephalitis virus and DENV, we decided to study whether GRP78 played a role in TMUV binding to the cells. The result of mass spectrometry are shown in **Figure 1D**. The interaction between TMUV and GRP78 was further confirmed by co-immunoprecipitation assay. As shown in **Figure 1C**, the anti-GRP78 antibody can specifically recognize the 70 kDa protein band.

**Table 1:** LC-MS/MS analysis of 70 kDa protein.

	Protein name	NCBI number	Mass (Da)
1	Succinate dehydrogenase [ubiquinone] flavoprotein subunit, mitochondrial [Mesocricetus auratus]	XP_005065480.1	73,444
2	78 kDa glucose-regulated protein [Mesocricetus auratus]	XP_012977507.1	72,416
3	Dihydropyrimidinase-related protein 2 isoform X1 [Mesocricetus auratus]	XP_005075293.1	73,584
4	Apoptosis-inducing factor 1, mitochondrial isoform X1 [Mesocricetus auratus]	XP_005076907.1	67,000
5	Prolyl 4-hydroxylase subunit alpha-1-like isoform 2 [Cricetulus griseus]	ERE87262.1	75,764
6	Dolichyl-diphosphooligosaccharide–protein glycosyltransferase subunit 1 [Mesocricetus auratus]	XP_005071196.1	68,395
7	Annexin A6 isoform X1 [Mesocricetus auratus]	XP_005067811.1	76,280
8	Transketolase [Mesocricetus auratus]	XP_005085264.1	68,342
9	Cytoskeleton-associated protein 4 [Mesocricetus auratus]	XP_012978803.1	60,111


### The Antibody Against GRP78 Competitively Inhibited TMUV Entry

**FIGURE 2:**
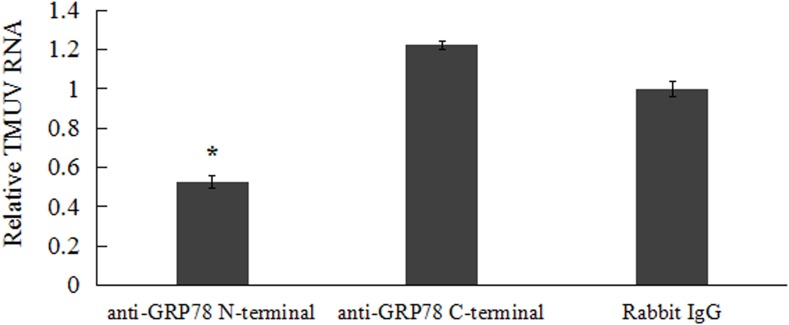
Anti-GRP78 antibody inhibits TMUV infection in BHK-21 cells. BHK-21 cells were pre-incubated with 100 μg/ml rabbit IgG, anti-GRP78 N-terminal or anti-GRP78 C-terminal antibodies at 4°C or 1 h followed by TMUV infection. The level of TMUV RNA in rabbit IgG incubated cells was taken as 1 for determining the relative RNA levels. The viral RNA was compared to those in rabbit IgG incubated cells. Data were presented from three independent experiments and statistic analysis was done with SPSS software. The asterisk designates statistically significant differences (*p* < 0.05) between groups.

An infection inhibition assay was then taken to further determine the role of GRP78 in TMUV entry. BHK-21 cells were pre-incubated with antibodies against the N- or C-terminal domains of GRP78, followed by TMUV infection as described in Section “Materials and Methods.” Viral RNA level was significantly lower in N-terminal GRP78 antibody treated cells compared with non-specific rabbit IgG treated cells, suggesting the N-terminal GRP78 antibody had a significantly reduced (50%) virus binding. However, the C-terminal GRP78 antibody failed to affect the virus binding (**Figure 2**).

### Expression of GRP78 on the Surface of BHK-21 Cells

**FIGURE 3:**
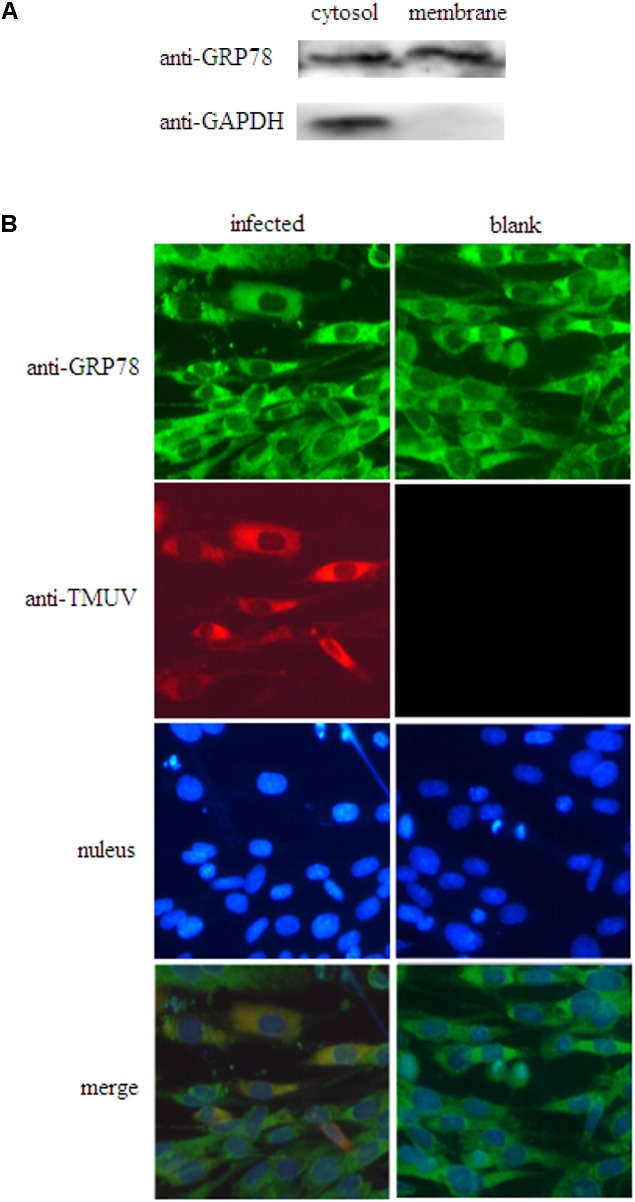
Colocalization of GRP78 and TMUV on the surface of BHK-21 cells. **(A)** Membrane proteins and cytosolic proteins of BHK-21 cells were western blotted with GRP78 antibody. GAPDH was chosen as cytosolic marker. **(B)** Immunofluorescence detection of non-permeabilized cells infected with TMUV.

Traditionally, GRP78 was regarded as an endoplasmic reticulum lumenal protein. Recent studies showed that GRP78 also expressed on the cell surface (Tsai et al., 2015). In order to validate the expression of GRP78 on the surface of BHK-21 cells, membrane proteins and cytosolic proteins from BHK-21 cells were isolated and subject to western blot analysis. The result of western blot showed that GRP78 can be expressed on the surface of BHK-21 cells (**Figure 3A**).

### GRP78 and TMUV Colocalized in BHK-21 Cells

Indirect immunofluorescent assay was used to observe colocalization of GRP78 and TMUV in BHK-21 cells. The non-permeabilized fixed cells were stained with anti-GRP78 antibody and monoclonal antibody against TMUV as primary antibodies followed by an Alexa-488-anti-rabbit and Alexa-594-anti-mouse as the secondary antibodies. Visualization under a fluorescent microscope showed that GRP78 colocalized with TMUV on the surface of BHK-21 cells (**Figure 3B**).

### RNA Interference With GRP78 in BHK-21 Cells Inhibited TMUV Infection

**FIGURE 4:**
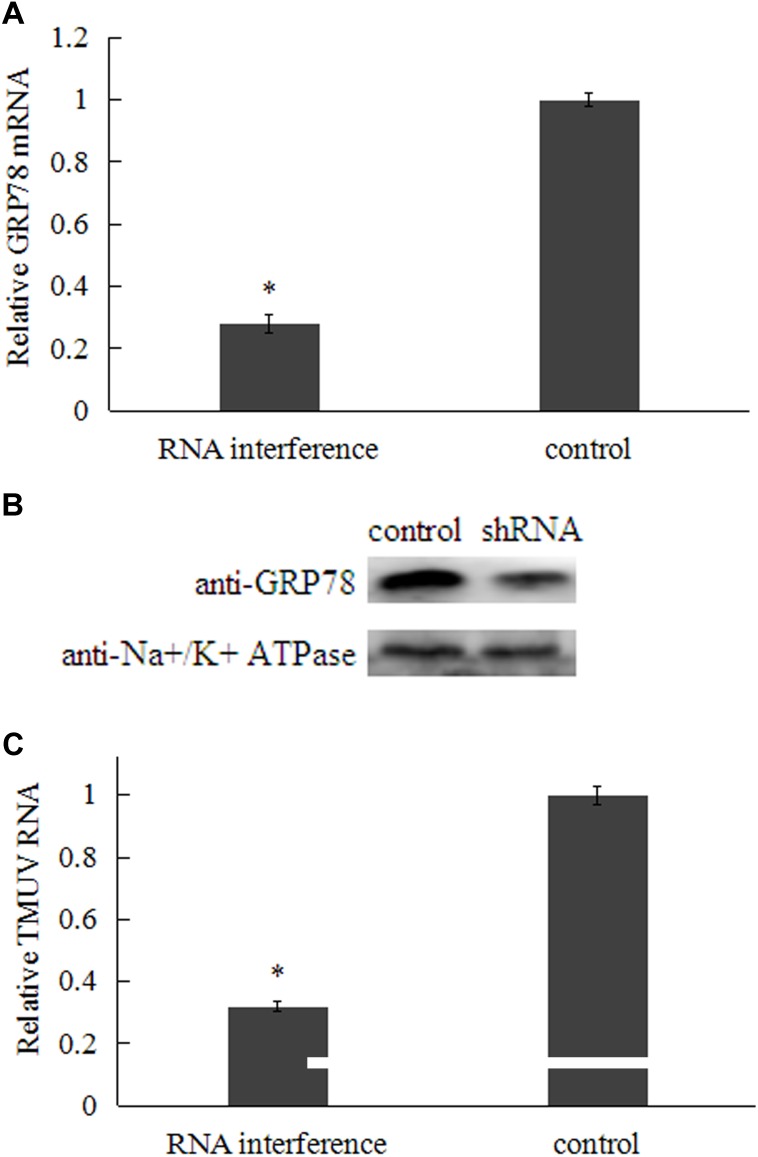
Effect of GRP78 shRNA transfection on TMUV entry at 48 h post-transfection. **(A)** Relative GRP78 mRNA level in transfected BHK-21 cells. **(B)** Analysis of surface-expressed GRP78 protein in transfected BHK-21 cells by western blot (Na+/K+ ATPase was chosen as plasma membrane marker). **(C)** Relative TMUV RNA were detected by qRT-PCR. Data were presented from three independent experiments and the asterisk designates statistically significant differences (*p* < 0.05) between groups.

To further demonstrate the role of GRP78 in TMUV binding and entry into cells, the protein was depleted by shRNA in BHK-21 cells. qRT-PCR indicated that the mRNA level of GRP78 was significantly inhibited in the BHK-21 cells (**Figure 4A**). Cell surface protein of transfected cells was purified and subjected to western blot analysis. The results of western blot showed that protein level of surface-expressing GRP78 decreased about 70% (**Figure 4B**). Compared to cells transfected with empty vector, the GRP78 shRNA-transfected cells showed a significant reduction (approximate 60%) in TMUV RNA levels (**Figure 4C**). These results suggest that GRP78 plays a significant role in the process of TMUV binding and entry into BHK-21 cells.

### GRP78 Over-Expression in BHK-21 Cells Improved TMUV Infection

**FIGURE 5:**
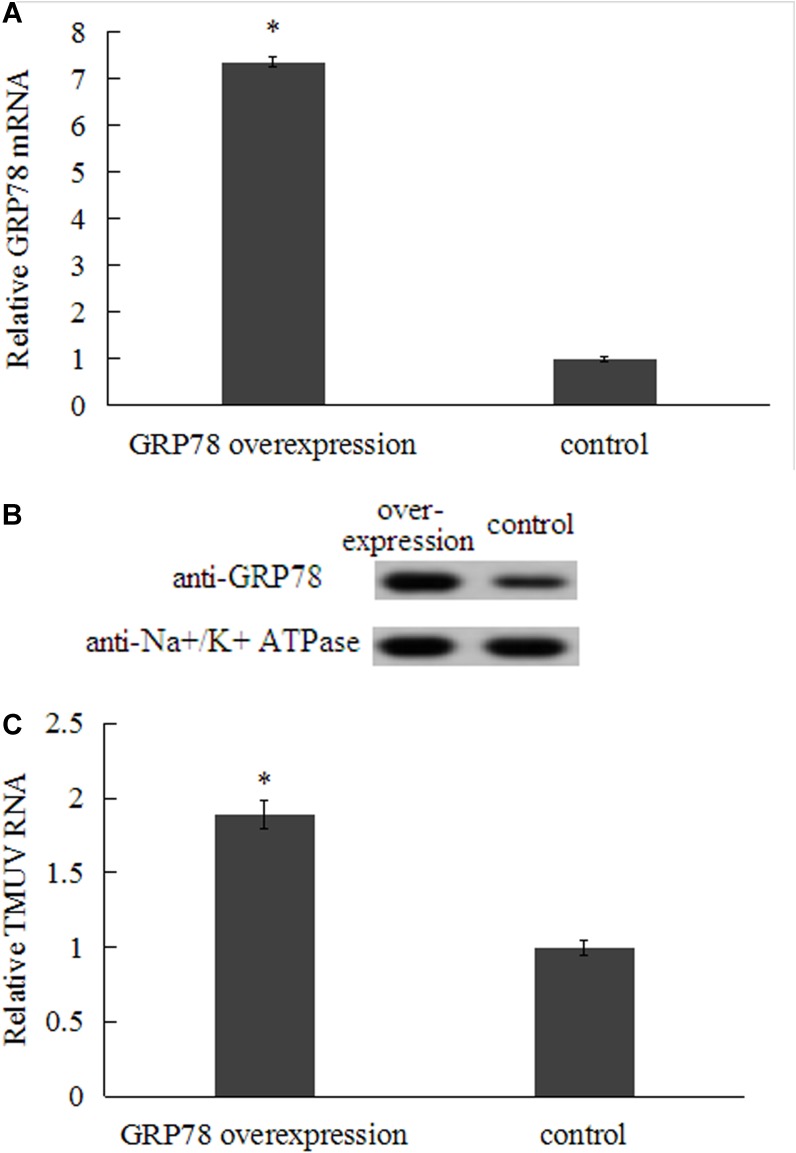
Effect of over-expression of GRP78 on TMUV entry at 48 h post-transfection. **(A)** qRT-PCR analysis of over-expression of GRP78 in transfected BHK-21 cells. **(B)** Surface-expressed GRP78 protein was determined by western blot (Na+/K+ ATPase was chosen as plasma membrane marker). **(C)** The TMUV entry in BHK-21 cells transfected with GRP78-pcDNA was measured by qRT-PCR. Data were presented from three independent experiments and the asterisk designates statistically significant differences (*p* < 0.05) between groups.

BHK-21 cells were transfected with GRP78-pcDNA and empty pcDNA3.1, respectively. At 48 h post-transfection, TMUV was added into transfected cells at an dose of 200 TCID_50_ and viral E gene was detected by qRT-PCR. Compared to cells transfected with empty pcDNA3.1, the cells transfected with GRP78-pcDNA showed a significant increase in expression of cell surface GRP78. Correspondingly, TMUV RNA levels in GRP78-pcDNA transfected cells increased significantly (**Figure 5**). It is demonstrated that over-expression of GRP78 can support TMUV uptake and entry into BHK-21 cells.

## Discussion

GRP78, a member of the Hsp70 family, is also referred to as Bip/HSPA5. The GRP78 protein consists of membrane-associated and trans-membrane segments (Reddy et al., 2003; Quinones et al., 2008; Gonzalez-Gronow et al., 2009). It can be found on the cell surface of various cell types including tumor cells, neural stem cells, spermatogenic cells, epidermal cells, arterial smooth muscles cells, monocytes, and B-cells (Mayer, 2005). More than an endoplasmic reticulum stress-regulating chaperone, GRP78 is associated multiple cellular functions, such as stress response, antigen processing, cancer, inflammatory, control of cell proliferation and autoimmune diseases (Liu et al., 2017).

Although GRP78 has been traditionally regarded as endoplasmic reticulum lumenal protein, now many studies provide the evidence that GRP78 can also be detected on cell surface and exerts functions beyond the endoplasmic reticulum (Tsai et al., 2015). Substantial research has found that GRP78 is a receptor or co-receptor for pathogens such as DENV (Wati et al., 2009), coxsackievirus (Triantafilou et al., 2002), herpes simplex virus (Wu et al., 2011), avian leukosis virus (Wang et al., 2016) and Japanese encephalitis virus (Nain et al., 2017). In this study, we identified GRP78 as a TMUV-binding protein in BHK-21 cells by the VOPBA, mass spectrometry analysis and co-immunoprecipitation assay. Western blot further validated that GRP78 was expressed on the surface of BHK-21 cells. Indirect immunofluorescent assay of non-permeabilized cells showed the colocalization between GRP78 and TMUV in BHK-21 cells. These results suggest that GRP78 is a putative receptor for TMUV.

GRP78, like other Hsp70 proteins, consists of two domains: the 44 kD N-terminal domain, which exhibits ATPase activity, and C-terminal domain, which contains a peptide-binding subdomain followed by helical and variable C-terminal tail (Chevalier et al., 2000). It is believed that C-terminal of GRP78 involves in protein binding, while N-terminal acts as regulatory domain governing binding through conformational changes of GRP78 (Jindadamrongwech et al., 2004). Antibody inhibition assay showed that TMUV binding was significantly inhibited when BHK-21 cells were blocked with polyclonal antibody against N-terminal of GRP78, while antibody against the C-terminal did not exhibit any blocking effect on infection. Interestingly, antibody against the C-terminal enhance infection slightly (**Figure 2**). We speculated that binding of C-terminal specific antibody induced the conformational changes of GRP78 which can increase the virus-receptor-binding ability. It is also possible that binding of C-terminal antibody activated the cells to improve replication of virus.

RNA interference and over-expression were employed to determine the role of GRP78 in TMUV attachment. In order to rule out the interference from GRP78 located in endoplasmic reticulum, cell surface protein was biotinylated and purified after 48 h post transfection and the levels of GRP78 expressed on the cell surface were determined by western blot. The results showed that levels of surface-expressed GRP78 was significantly knocked down or enhanced by RNA interference and over-expression of GRP78, respectively. Previous studies showed that flavivirus replication complexes are established in the infected cell by 6–8 h post inoculation (Sharma et al., 2014; Nain et al., 2017). Therefore, to analyze the role of GRP78 in TMUV binding and entry into cells, and rule out the potential effect of endoplasmic reticulum stress and unfolded protein response induced by flavivirus, we detect the viral RNA at 3 h post inoculation. The results demonstrated that knock-down of surface-expressed GRP78 by shRNA impaired virus attachment and entry. Furthermore, the over-expression of GRP78 on the surface of BHK-21 cells improved the entry of TMUV, which suggests that GRP78 is involved in viral attachment and entry into BHK-21 cells.

Considering flavivirus may use multiple receptors for cell entry and the binding between flaviviruses and its cellular receptors is a multistep process (Liu et al., 2017), the GRP78 antibody did not completely abolish the virus infection, it is highly likely that additional molecules besides GRP78 act as TMUV receptor. Indeed, HSPA9 has been reported to be involved in TMUV entry into DF-1 cells (Liu et al., 2017). Thus, further studies on the interactions between GRP78 and TMUV are undergoing to better understand the receptor system and infection mechanism of TMUV. Meanwhile, recognition of other receptors or co-receptors involved in TMUV infection system is also under way in our laboratory.

In summary, this is the first report that identified GRP78 as receptor for TMUV in BHK-21 cells. GRP78 is involved in TMUV receptor binding and entry to BHK-21 cells, indicating its important role in virus infection. The identification of GRP78 will allow us to better understand the mechanism of TMUV pathogenesis. Furthermore, the multiple critical roles of GRP78 on the infection of TMUV may provide novel target for anti-viral drug design and additional information regarding TMUV prevention and control.

## Author Contributions

DZ and YiL designed the experiments. DZ, QL, KH, and HW performed the experiments. JY, KB and YuL analyzed the data. NL and YT prepared the reagents. DZ wrote the manuscript.

## Conflict of Interest Statement

The authors declare that the research was conducted in the absence of any commercial or financial relationships that could be construed as a potential conflict of interest.
